# Defective STAT1 activation associated with impaired IFN-γ production in NK and T lymphocytes from metastatic melanoma patients treated with IL-2

**DOI:** 10.18632/oncotarget.8683

**Published:** 2016-04-11

**Authors:** Geok Choo Sim, Sheng Wu, Lei Jin, Patrick Hwu, Laszlo G. Radvanyi

**Affiliations:** ^1^ Department of Melanoma Medical Oncology, University of Texas MD Anderson Cancer Center, Houston, TX 77030, USA; ^2^ Department of Immunology, Moffitt Cancer Center, Tampa, FL 22612, USA

**Keywords:** melanoma, high-dose IL-2, STAT1, IFN-γ, NK cells

## Abstract

High dose (HD) IL-2 therapy has been used for almost two decades as an immunotherapy for metastatic melanoma. IL-2 promotes the proliferation and effector function of T and NK cells through the tyrosine phosphorylation and activation of signal transducer and activator of transcription factors (STAT), especially STAT5. However, whether any defects in STAT activation exist in T and NK lymphocytes from melanoma patients are under debate. Here, we measured the extent of HD IL-2-induced phosphorylation of STAT5 and STAT1 in lymphocyte subsets from metastatic melanoma patients and healthy controls at a single cell level using flow cytometry. We found no defects in IL-2-induced STAT5 phosphorylation and induction of proliferation in T and NK cell subsets *in vitro*. This was confirmed by measuring *ex vivo* STAT5 activation in whole blood collected from patients during their first bolus HD IL-2 infusion. IL-2 also induced STAT1 phosphorylation via IFN-γ receptors in T and NK cell subsets through the release of IFN-γ by CD56^hi^ and CD56^lo^ NK cells. Further analysis revealed that melanoma patients had a sub-optimal STAT1 activation response linked to lower IL-2-induced IFN-γ secretion in both CD56^hi^ and CD56^low^ NK cell subsets. STAT1 activation in response to IL-2 also showed an age-related decline in melanoma patients not linked to tumor burden indicating a premature loss of NK cell function. Taken together, these findings indicate that, although STAT5 activation is normal in metastatic melanoma patients in response to IL-2, indirect STAT1 activation is defective owing to deficiencies in the NK cell response to IL-2.

## INTRODUCTION

Melanoma is one of the most rapidly increasing forms of cancer with more than 70,000 people are estimated to be diagnosed with melanoma in the United States in 2015 [1]. Metastatic melanoma has been found to be more resistant to chemotherapy and radiation therapy than other major forms of cancer such as epithelial cancers and sarcomas and, as a result, much effort has gone into developing alternative treatment approaches, such as immunotherapy using cytokines, antigen-specific vaccination, and adoptive T-cell therapy to activate the immune system [2–4]. This interest in immunotherapy also stems from the fact that melanoma has been found to be quite an immunogenic form of cancer with most patients having brisk infiltration of lymphocytes inside and around metastatic tumor beds. Activation of T cells and natural killer (NK) cells both systemically and locally within the tumor microenvironment using cytokines such as interleukin-2 (IL-2) is one approach that has been used extensively against metastatic melanoma.

High dose (HD) IL-2 therapy has been used as an effective immunotherapy against melanoma for about 20 years. IL-2 binds to IL-2 receptors at different affinities, with intermediate affinity binding to the IL-2Rbg heterodimer form of IL-2Rβγ heterodimer and high affinity binding to the IL-2Rαβγ trimeric complex [5]. The β subunit triggers signaling by undergoing autophosphorylation of key tyrosines in cytoplasmic domain in response to IL-2 that induces the binding of a tyrosine kinases of the Janus kinase (JAK) (JAK2 and JAK3) family via SH2 domain interactions [6–8].

HD IL-2 as an intermittent bolus infusion therapy has been tested in clinical trials for different tumor types [9–11] and is FDA-approved for the treatment of metastatic renal cell carcinoma and melanoma [8, 12, 13]. The standard regimen involves bolus infusion of 600,000 to 720,000 IU/kg intravenously every 8 hours for up to 14-15 doses or until tolerance per treatment cycle, with treatment cycles of therapy at 2-3 weeks interval [14–16]. Currently, the objective clinical response rate for stage IV melanoma ranges in 10-16% [12]. However, if the metastases are located only in cutaneous or subcutaneous sites, response rates could be as high as 50% [17]. To increase the response rates, HD IL-2 treatment has been combined with other treatments, such as adoptive cell transfer (ACT) therapy [18].

Because of the pleiotropic nature of the effects of IL-2, the precise mechanisms of IL-2 anti-tumor effects still remained unclear. Animal models showed that the HD IL-2 therapy activates and expands NK cells and CD8^+^ T cells mediating anti-tumor responses [19]. NK cells have been found to be especially sensitive to IL-2 and animal tumor model studies have found a dependence on activated NK cells in the induction of anti-tumor effects [20, 21]. However, there is also considerable evidence demonstrating that IL-2 can expand CD4^+^CD25^+^Foxp3^+^ T-regulatory cells [15, 22] and program mature T cells for apoptosis [23]. So both positive and negative effects are elicited by IL-2. An overall defect in the function of the immune system in cancer patients is another mechanism that may reduce the efficacy of IL-2 therapy. For example, the T cell receptor (TCR) zeta-chain has been shown to be down-regulated in T cells of melanoma patients [24, 25], while in NK cells the expression of NKp30, NKp46 and NKG2D has been found to be reduced in some patients [26, 27]. At present, little is known about whether any defects exist in the response of lymphocytes from metastatic melanoma patients to HD IL-2 despite its use in the clinic now for about two decades. Differences in the sensitivity or response of specific lymphocyte subsets between different individuals may account for why only a fraction of patients with non-cutaneous visceral metastases respond to HD IL-2 therapy with an objective tumor shrinkage.

A recent study reported that both tyrosine phosphorylation of signal transducer and activator of transcription 5 (STAT5) and STAT1 in response to IL-2 stimulation were impaired in melanoma patients and this was correlated with increasing clinical stage [28]. Other recent studies have examined the response of T and NK lymphocytes from melanoma patients to type I and type II interferons (IFN), where a defective activation of STAT1 in response to IFN-α and IFN-γ was found in some lymphocyte subsets relative to healthy controls [29, 30]. Another study found that STAT5 phosphorylation in peripheral blood lymphocytes following IL-2 therapy and prolonged STAT5 activation after IL-2 therapy may correlate to positive clinical responses [31]. These and other related recent studies have used an intracellular flow cytometry staining approach to monitor intracellular STAT activation measuring the extent of phosphorylation of key tyrosine residues in STAT5 and STAT1 at single cell level. This technique is combined with cell surface staining to distinguish how STATs are activated in different T-cell and NK cell subsets.

In this study, we measured the extent of STAT5 and STAT1 activation, proliferative capacity and cytokine production in different peripheral blood lymphocyte subsets from a group of patients with metastatic melanoma in comparison to a cohort of healthy controls upon HD IL-2 stimulation. Our data indicated that dysregulated IFN-γ secretion by NK cells contributed to a significant defect in STAT1 but not STAT5 activation in patients with advanced melanoma in response to IL-2 stimulation. These findings also suggest that a generalized suppression of STAT1 signaling pathway may be involved in the lack of therapeutic benefit of HD IL-2 in most patients with melanoma.

## RESULTS

### HD IL-2-induced STAT5 activation is not impaired in different NK and T cell subsets from patients with metastatic melanoma

**Figure 1:**
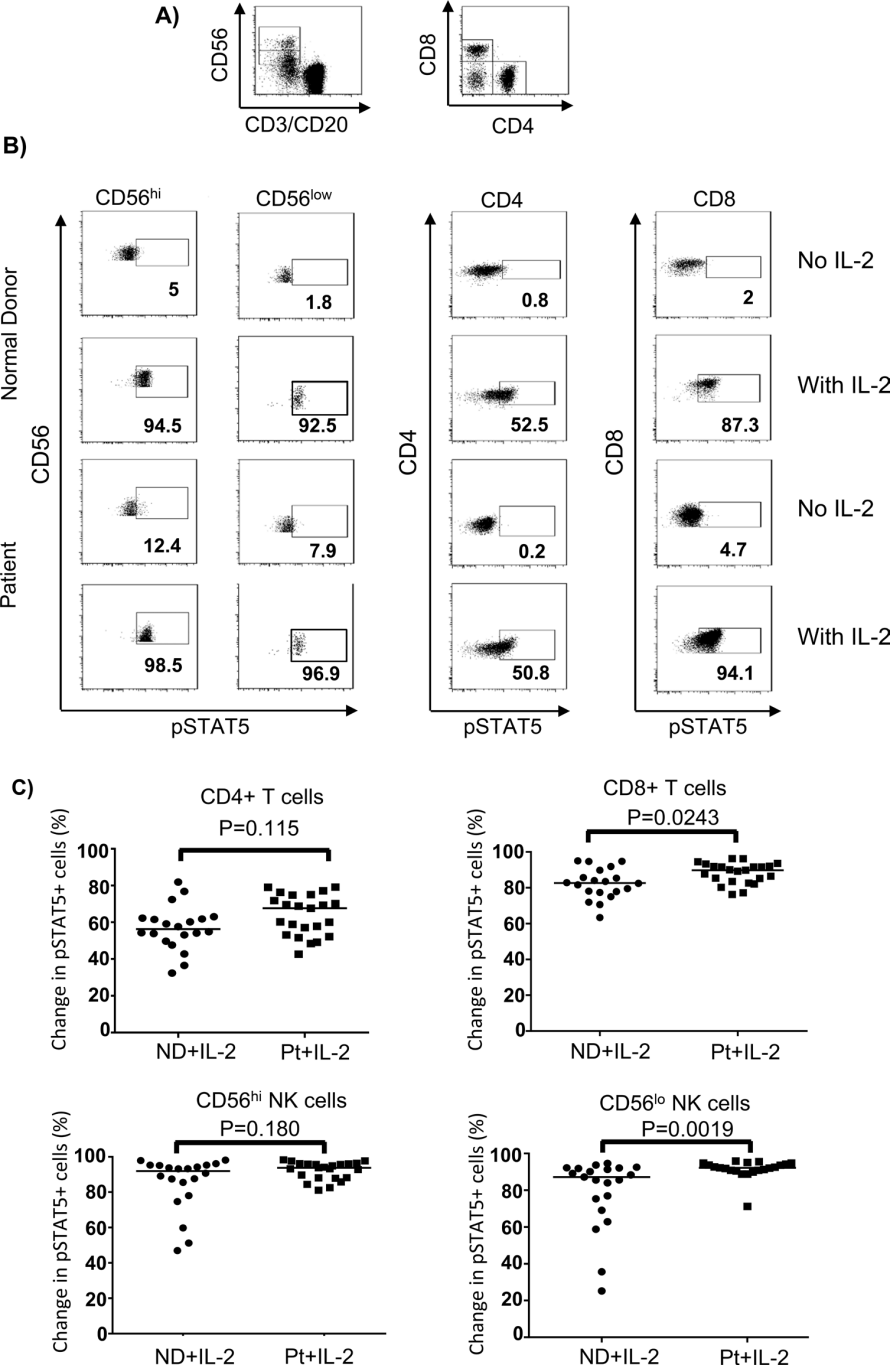
HD IL-2-induced STAT5 activation is not impaired in different NK and T cells from patients PBMC (2 × 10^6^) from age and gender-matched healthy controls (ND) and patients with stage IV melanoma (Pt) were treated with or without 6,000 IU/ml IL-2 for 24 hours. Cells were harvested and stained for surface markers followed by intracellular pSTAT5 staining. **A.** Dot plots show the gating strategy for identifying CD4^+^T, CD8^+^ T cells, CD56^hi^ and CD56^lo^ NK cells in PBMC. B cells were excluded from analysis by gating out anti-CD20 expressing cells. **B.** Representative flow cytometry dot plots from one healthy control and one melanoma patient show the HD IL-2-induced STAT5 activation were intact in CD4^+^ T cells, CD8^+^ T cells, CD56^hi^, and CD56^lo^ NK cells subsets. **C.** Scatter plots show change of pSTAT5^+^ cells in the indicated IL-2 stimulated lymphocyte subsets from healthy controls and patients were calculated by subtracting the frequency of pSTAT5^+^ cells of non-stimulated from IL-2 stimulated cells. The median of each data set in scatter plots are indicated by the horizontal bars. Two-sided Mann-Whitney test was used to compare values from cancer patients with age-matched healthy controls.

Previous studies have suggested that melanoma patients had multiple defects in their immune systems induced by tumor cells [24, 27–30, 32] that may facilitate resistance to immunotherapy, such as with HD IL-2. We measured activation of STAT signaling molecules in different lymphocyte subsets from a total of 23 patients with metastatic melanoma (stage III and IV) and 23 healthy controls in response to HD IL-2 stimulation at single cell level using intracellular staining and flow cytometry approach (also called “phosphoflow”). We first analyzed the extent of HD IL-2 induced STAT5 phosphorylation (pSTAT5) in different lymphocyte subsets. Phosphoflow staining of pSTAT5 in IL-2 stimulated peripheral blood mononuclear cells (PBMC) from healthy controls was initially performed to identify the optimum dose of IL-2 in activating lymphocyte subpopulations (Figure S1 on-line). Based on the dose-response curve and the dose given during clinical treatment, 6,000 IU/ml IL-2 was found to be the optimal dose and was used in all of the subsequent experiments. Next, PBMC from age and gender-matched healthy controls and patients with metastatic melanoma (Table S1 on-line) were stimulated with 6,000 IU/ml IL-2 for 24 hours followed by pSTAT5 phosphoflow staining. Experiments with PBMC from normal donors found that IL-2 induced a rapid induction of STAT5 tyrosine phosphorylation after 20 mins of stimulation *in vitro* which was stable for 24 h (data not shown). Without IL-2 treatment, pSTAT5 activations in all of the T and NK lymphocyte subsets from both healthy controls and patients were remained at basal level after 24 h of treatment (Figure 1A and 1B). However, CD4^+^ T cells, CD8^+^ T cells and both CD56^hi^ and CD56^lo^ NK cell subsets from healthy controls and patients responded to HD IL-2 stimulation with dramatic increase of pSTAT5 activation (Figure 1B). As shown in Figure 1C, when PBMC from healthy controls were treated with HD IL-2, there were 91.9% CD56^hi^, 87.6% CD56^low^ NK cells and 82.7% CD8^+^ T cells showed pSTAT5 expression, while only 56.3% CD4^+^ T cells expressed pSTAT5. There was no significant reduction in the proportion of pSTAT5-expressing T and NK cell subpopulations from patients as compared to healthy controls. However, we observed an increase of pSTAT5-activation in CD8^+^ T cells and CD56^low^ NK cells from patients when stimulated with HD IL-2 (Figure 1C).

**Figure 2:**
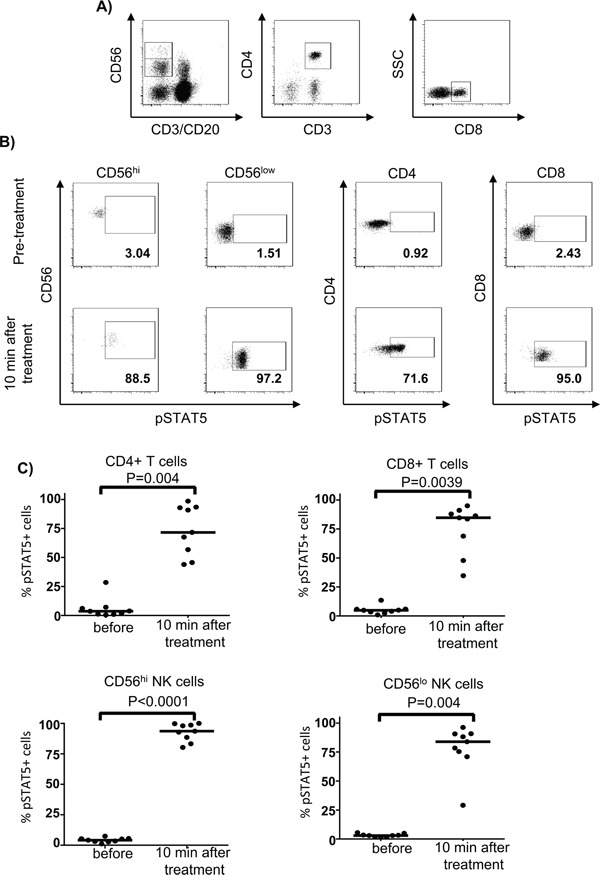
First bolus of IL-2 treatment induced a dramatic STAT5 activation in whole blood from patients Heparinized fresh whole blood was obtained from patients with stage IV melanoma (n=9) before or 10 min after HD IL-2 treatment. Whole blood samples were directly fixed/permeabilised for pSTAT5 phosphoflow staining. **A.** The *ex-vivo* STAT5 activation was measured in CD4^+^ T, CD8^+^ T cells, CD56^hi^ and CD56^lo^ NK cells and the respective cell subsets were identified by the gating strategy shown in the representative dot plots. **B.** Flow cytometry dot plots and **C.** scatter plots show that the HD IL-2 induced STAT5 activation exhibit a similar pattern in all the T and NK cell subsets that were studied before and 10 min after first bolus of IL-2 treatment. The median of each data set in scatter plots are indicated by the horizontal bars. Two-sided Mann-Whitney test was used to compare the values from cancer patients with age-matched healthy controls.

To confirm that T and NK cell subsets from patients have intact pSTAT5 activation and to exclude possible effects of blood cell processing and cell culture on STAT signaling, we also investigated *ex vivo* pSTAT5 levels in different lymphocyte subsets in whole blood samples before and 10 min after beginning the first HD IL-2 infusion (15 min total infusion time) in previous IL-2-naïve patients. Phophosflow staining data showed dramatic STAT5 activation in different T and NK cell subsets from freshly collected blood samples and exhibited a similar pattern as that in isolated PBMC when stimulated with HD IL-2 at *in vitro* setting (Figure 2A, 2B and 2C).

### HD IL-2-induced a similar proliferation capacity in T and NK cell subpopulations from both normal controls and patients

**Figure 3:**
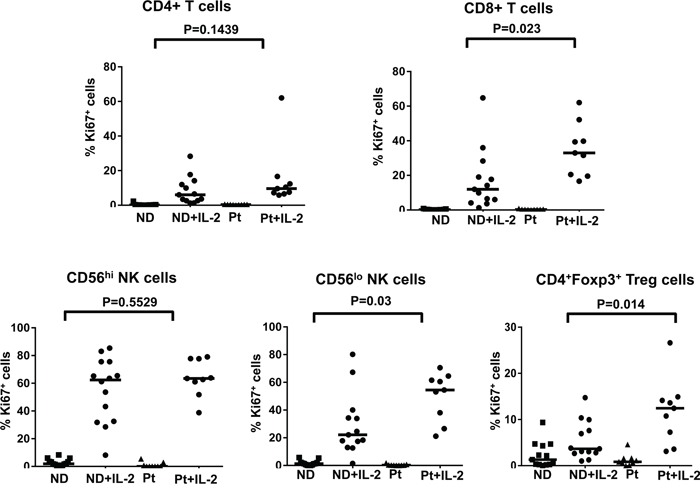
No defects in the proliferation capacity of IL-2-treated T and NK cell subsets from patient The proliferation capacity of different T and NK cell subsets were measured by the expression of Ki67. Scatter plots shown are frequency of Ki67 expressing cells in various T and NK subpopulations in PBMCs (2 × 10^6^) from age and gender-matched healthy controls (ND) (n=17) and patients (Pt) (n=9) with stage IV melanoma with or without 6,000 IU/ml IL-2 stimulation for 4 days. Comparison of the proportion of Ki67 expressing cells in CD4^+^ T cells, CD8^+^T cells, CD56^hi^ NK cells, CD56^lo^ NK cells, and CD4^+^Foxp3^+^Tregs between patients and healthy donors. The median of each data set in the scatter plots are indicated by the horizontal bars. ANOVA test was used to compare the values from cancer patients with age-matched healthy controls.

Because pSTAT5 has been shown to regulate lymphocyte proliferation [33], our data indicating normal STAT5 phosphorylation in the lymphocyte subsets from melanoma patients compared to healthy controls prompted us to measure the proliferation capacity of these cell subsets in response to HD IL-2 stimulation as a functional readout of IL-2 activity. We performed intracellular staining for Ki67 4 days after IL-2 treatment. Ki67 is expressed by cycling cells with the level of expression correlating with *in vivo* DNA labeling [34]. HD IL-2 treatment induced a marked increase in the proportion of Ki67-expressing cells in all T and NK cell subsets from both healthy controls and patients. There was no significant reduction in the proliferation capacity of CD4^+^, CD4^+^Foxp3^+^ T regulatory cells (Treg), CD56^hi^ and CD56^low^ NK cells from melanoma patients as compared to healthy controls (Figure 3 and Figure S2 on-line). However, we observed a higher proportion of CD8^+^Ki67^+^ T cells and CD56^low^ Ki67^+^ NK cells from patients than healthy controls (*p* < 0.05) (Figure 3) and this was correlated with a higher proportion of pSTAT5-expressing CD8^+^ T and CD56^low^ cells in patients (Figure 1C). Taken together, there was no evidence to suggest that STAT5 activation in T and NK cell populations from patients was significantly inhibited in response to HD IL-2 treatment.

### Secretion of IFN-γ by different lymphocyte subsets is defective in melanoma patients

**Figure 4:**
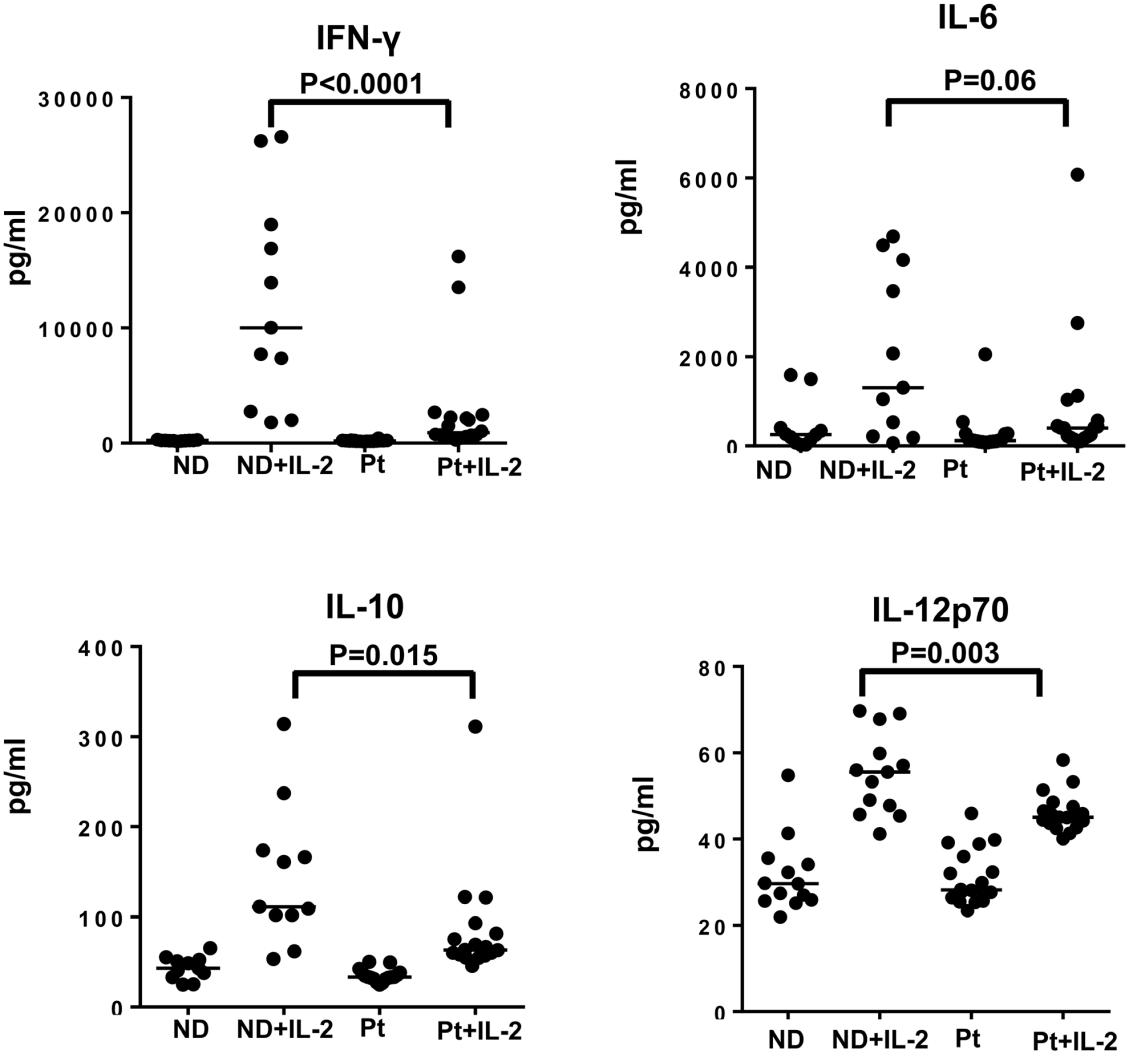
Secretion of IFN-γ by different lymphocyte subsets is defective in melanoma patients Cytokine secretion by PBMC from healthy controls (ND) and melanoma patients (Pt) was measured for investigating the functional properties of T and NK cell subsets in response to HD IL-2 treatment. PBMCs (2 × 10^6^) from melanoma patients (n=20) and healthy donors (n=15) were stimulated with 6,000 IU/ml IL-2 for 24 h and supernatant were harvested and analyzed for the production of IFN-γ, IL-12p70, IL-10 and GM-CSF using MSD multiplex cytokine assay. Results show both IFN-γ and IL-12p70 were produced at lower levels by PBMC from melanoma patients than healthy controls. Horizontal bars in each data set shown in scatter plots indicate median value of cytokine levels. Results are reported as concentration of cytokine release in HD IL-2 stimulated PBMCs. ANOVA test was used to compare the values from cancer patients with age-matched healthy controls.

Although our previous data showed no defect in the proliferation capacity and pSTAT5 activation of different lymphocyte subsets from melanoma patients compared to healthy controls, there could be impairment in other IL-2-induced effector functions, such as inflammatory cytokine production. PBMC from both patients and healthy controls were stimulated with HD IL-2 for 24 hours and culture supernatants were collected and measured for the levels of pro-inflammatory cytokines including IFN-γ, IL-12p70, GM-CSF, IL-10, TNF-α, IL-1β, IL-8 and IL-6. In response to HD IL-2 stimulation, IFN-γ (median: 10,006 in normal donors versus 896.2 pg/ml in patients) and IL-10 levels (median: 111.3pg/ml in normal donors versus 63.27 pg/ml in patients) were significantly reduced in PBMC from patients as compared to healthy controls (Figure 4 and Figure S3 on-line). There was a trend of lower IL-12p70, IL-6 GM-CSF, and TNF-α, production by PBMC from melanoma patients (Figure 4 and Figure S3 on-line). Since there was about 11-fold reduction in IFN-γ release while only 3-fold reduction in IL-6 secretion were observed, this indicated that a lack of IFN-γ production may be the major defect in PBMC from melanoma patients in response to HD IL-2.

### STAT1 activation upon IL-2 stimulation is impaired in lymphocytes of melanoma patients

**Figure 5:**
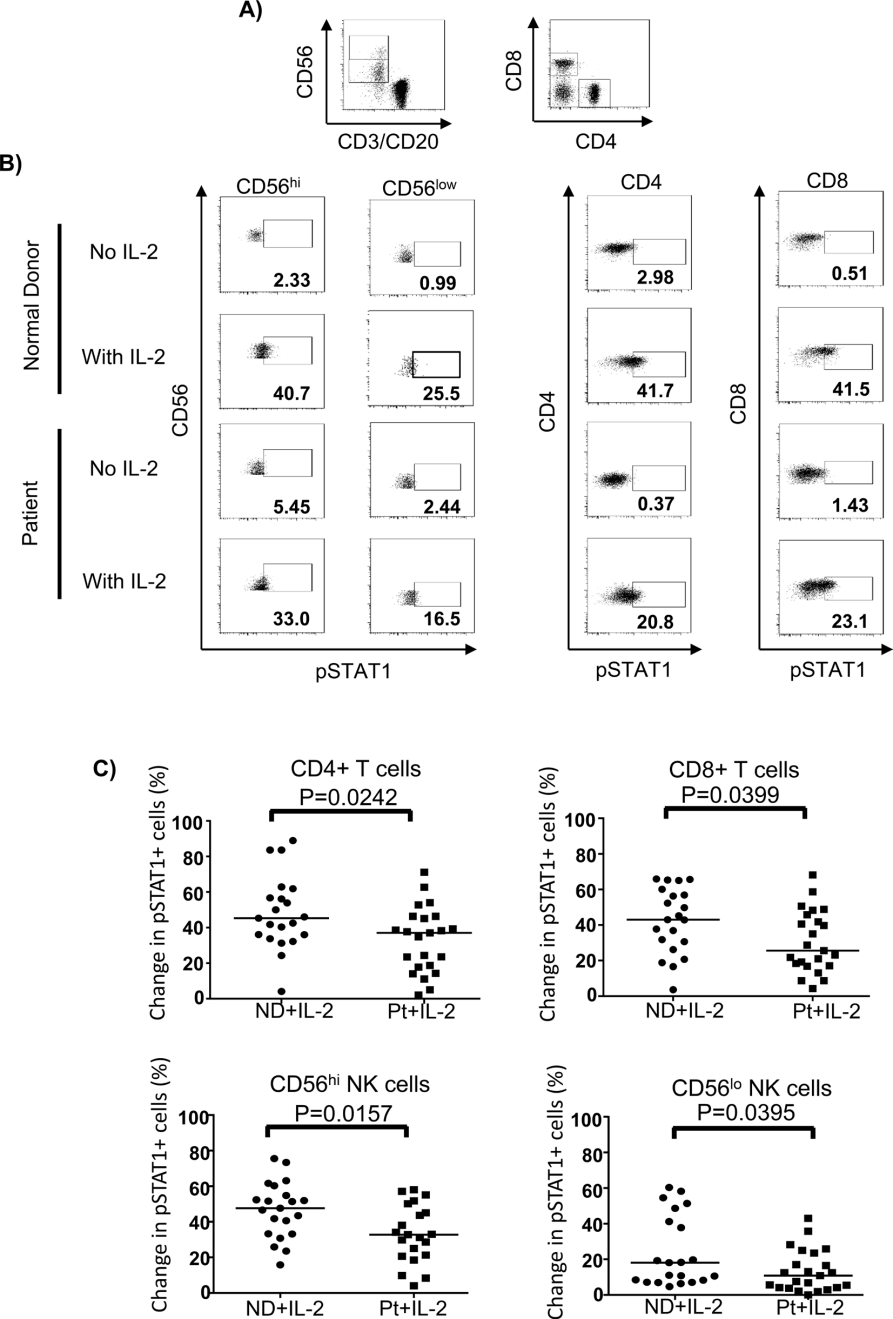
STAT1 activation upon IL-2 stimulation is impaired in lymphocytes of melanoma patients PBMC (2 × 10^6^) were treated with 6,000 IU/ml IL-2 or unstimulated for 24 hours and pSTAT1 was stained and measured by phosphoflow staining in patient (Pt) (N=23) and healthy controls (ND) (N=21). **A.** CD4^+^, CD8^+^ T cells and CD56^hi^ and CD56^lo^ NK cells are gated and shown in the dot plots. **B.** Representative flow cytometry dot plots of the HD IL-2 induced pSTAT5 activation in CD4^+^ T, CD8^+^ T cells and both CD56^hi^ and CD56^lo^ NK cells are shown for one healthy control and one melanoma patient. **C.** Comparison of the change in pSTAT1 in CD4^+^, CD8^+^ T cells and CD56^hi^ and CD56^lo^ NK cells between healthy controls and patients. The change in pSTAT1 was calculated by subtracting the frequency of IL-2 induced pSTAT1^+^ cells of non-stimulated from IL-2 stimulated cells.

**Figure 6:**
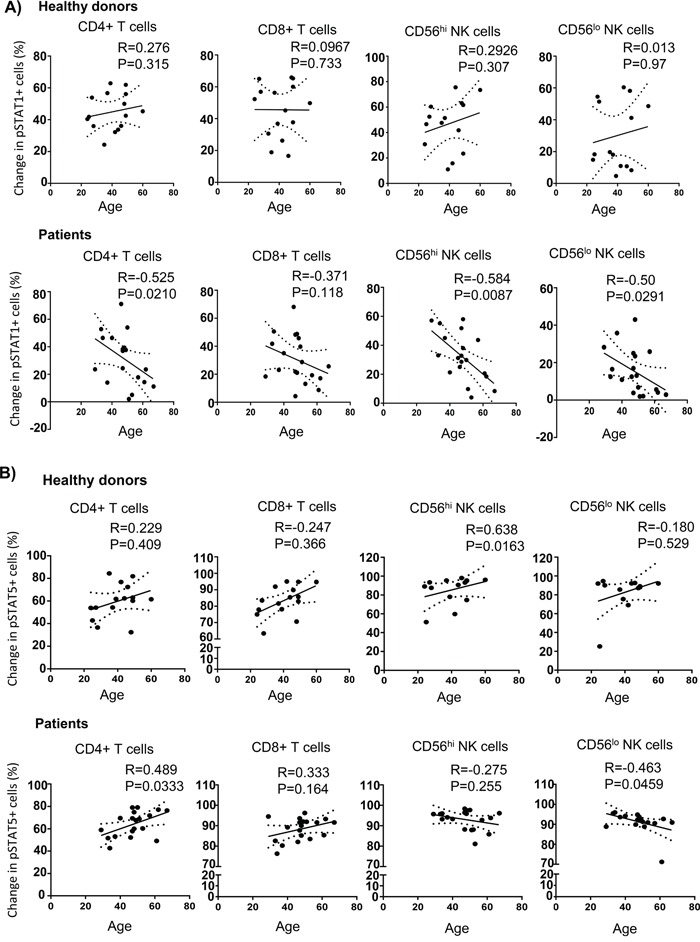
Age-associated STAT1 and STAT5 activation defects in melanoma patients Experiments were performed and analysed using phosphoflow as mentioned in materials and methods. Spearman correlation test was performed to analyze the correlation of aging the change of IL-2 induced pSTAT1 **A.** and pSTAT5 **B.** in CD4^+^ T cells, CD8^+^ T cells, and both CD56^hi^ and CD56^lo^ NK cells from healthy controls and melanoma patients. Means are indicated by the horizontal bars in each scatter plot. Spearman correlation test were used to compare the values from cancer patients with age-matched healthy controls.

STAT1 is a downstream transcription factor activated by the IFN-γ signaling pathway [35, 36]. Therefore, we hypothesized that phosphorylation of STAT1 in lymphocytes might be affected due to an impairment of IFN-γ release. To test this hypothesis, we tracked pSTAT1 activation in CD4^+^ and CD8^+^ T cells as well as CD56^hi^ and CD56^lo^ NK cells in response to IL-2. We found that melanoma patients exhibited significantly lower IL-2-induced pSTAT1 level than healthy controls in T and NK cell subsets (Figure 5A, 5B and 5C). This was not due to a higher median age of the melanoma patients tested or differences in gender (Table S2 and Table S3 on-line). However, when we plotted pSTAT1 staining level in association with age, we observed a significant inverse correlation in pSTAT1-expressing CD4^+^ T cells, CD56^hi^ and CD56^low^ NK cells, but not CD8^+^ T cells with age in melanoma patients but not healthy controls (Figure 6A). We also observed an inverse correlation of the proportion of pSTAT5^+^CD56^lo^ NK cells with aging in the melanoma patients in response to IL-2 with age, whereas the proportion of pSTAT5 expressing CD4^+^ T cells was positively associated with aging (Figure 6B). These age-related decreases of the IL-2-induced pSTAT5 induction were not observed in healthy controls (Figure 6B). We further examined whether an age-related reduction of pSTAT1-expressing immune cells was correlated with tumor burden and found no significant correlation between aging and lactate dehydrogenase (LDH) levels, a surrogate measure of tumor burden in metastatic melanoma patients (Figure S4).

To determine whether the defect in STAT1 activation was caused by a decreased expression of total intracellular STAT1 protein, we analyzed the expression of total STAT1 in CD4^+^, CD8^+^ T cells, CD56^hi^ and CD56^low^ NK cells using flow cytometry. The level of total STAT1 in CD4^+^, CD8^+^ T cells, CD56^hi^ and CD56^low^ NK cells were not dramatically different in patients versus the healthy controls. Although statistically significant, there was only a very subtle difference of total STAT1 levels in CD56^hi^ NK cells between patients and healthy controls (Figure S5A and Figure S5B on-line). We also pre-treated PBMCs with TGF-β before IL-2 stimulation, but no significant inhibition was found on the STAT1 activation or IFN-γ secretion (Figure S6 on-line). These data indicate that the phosphorylation of STAT1 is impaired in CD4^+^ T cells and CD56^hi^ and CD56^low^ NK cell subsets in response to IL-2 treatment and this defect is age-related but not likely due to a reduced proportion of total cells exhibited STAT1 activation in metastatic melanoma patients.

### Blockade of IFN-γ inhibits pSTAT1 activation in T and NK cell subsets

**Figure 7:**
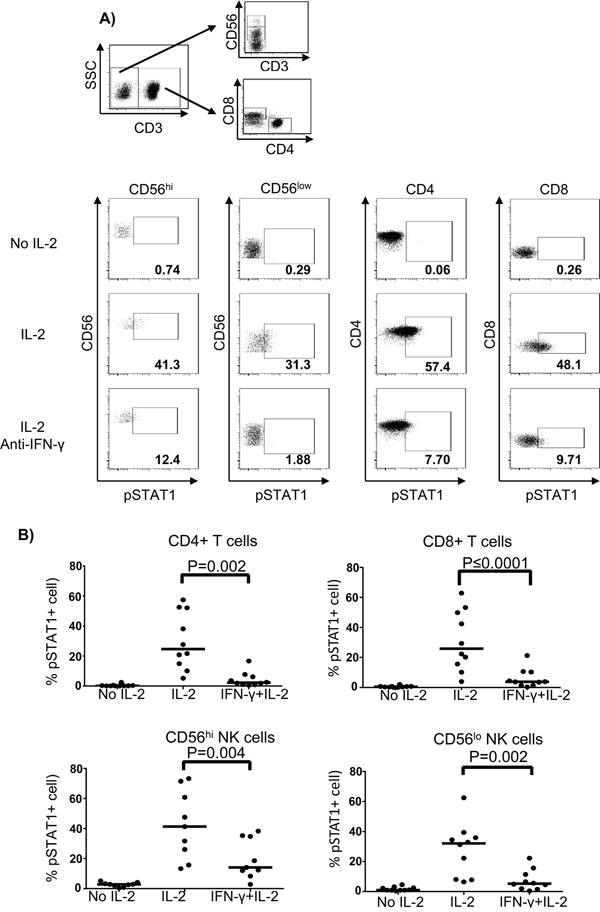
Blockade of IFN-γ inhibits the activation of pSTAT1 in T and NK cells PBMC (2 × 10^6^) from age and gender-matched healthy controls (ND) were treated with or without 6,000 IU/ml IL-2 for 24 hours. Neutralizing anti-IFN-γ mAb was added together with IL-2 at a final concentration of 100 μg/ml. Cells were then harvested and stained for pSTAT1 in T and NK cell subpopulations using phosphoflow approach. **A.** The gating strategy for identifying CD4^+^ T cells, CD8^+^ T cells, and CD56^hi^ and CD56^lo^ NK cells is shown in the dot plots. **B.** Representative dot plots from one healthy donor show the pSTAT1 expression in CD4^+^ T cells, CD8^+^ T cells and both CD56^hi^ and CD56^lo^ NK cells when treated with or without HD IL-2 in the presence or absence of neutralising anti-IFN-γ mAb. Comparison of the percentage of HD IL-2 induced pSTAT1 activation in CD4^+^ T cells, CD8^+^ T cells and CD56^hi^ and CD56^lo^ NK cells between healthy donors and patients. The medians are indicated by the horizontal bars. ANOVA test was used to compare the values from cancer patients with age-matched healthy controls.

To test our hypothesis that IFN-γ mediated pSTAT1 activation in response to IL-2 stimulation, we performed IFN-γ blockade in normal donor PBMC treated with HD IL-2. The IL-2-induced pSTAT1 level was greatly inhibited in all lymphocyte subpopulations by IFN-γ blockade using an added anti-IFN-γ mAb (Figure 7A and 7B). In contrast, IFN-γ blockade in the presence of HD IL-2 resulted in elevated pSTAT5 levels in CD4^+^ and CD8^+^ T cells but not in CD56^hi^ and CD56^low^ NK cells as compared to HD IL-2 stimulation only (Figure S7A and S7B on-line). These experiments further suggested an autocrine or paracrine effect of IFN-γ on T and NK cells where secreted IFN-γ in response to IL-2 lead to activation of pSTAT1 in T and NK. Thus, an insufficient secretion of IFN-γ by PBMC from melanoma patient in response to HD IL-2 treatment could lead to defective pSTAT1 activation.

### NK cells predominantly secrete IFN-γ in response to HD IL-2 stimulation

**Figure 8:**
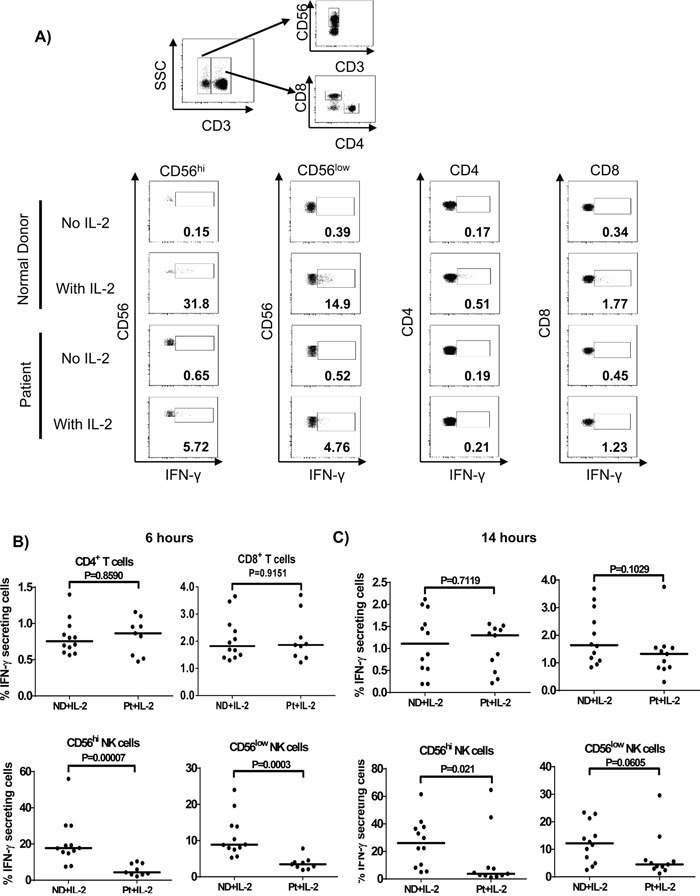
NK cells predominantly secrete IFN-γ in response to HD IL-2 stimulation Intracellular cytokine staining was performed on PBMCs (2 × 10^6^) from normal controls (ND) and patients (Pt) after incubated with HD IL-2 at 6,000 IU/ml for either 6 or 14 hrs. **A.** The secretion of IFN-γ was measured in CD4^+^ T, CD8^+^ T cells and both CD56^hi^ and CD56^lo^ NK cells from patients and healthy controls. Flow cytometry are shown for one representative patient and one healthy donor. Both CD56^hi^ and CD56^lo^ NK cells are shown to be the major lymphocyte subsets that produce IFN-γ after **B.** 6 h or **C.** 14 h of HD IL-2 stimulation. Medians are indicated by the horizontal bars in each data set. Two-sided Mann-Whitney test was used to compare the values from cancer patients with age-matched healthy controls.

To further investigate which lymphocyte subsets in melanoma patients was impaired in the secretion of IFN-γ, intracellular staining was performed on PBMC after 6 and 14 h of HD IL-2 stimulation (Figure 8A). The IFN-γ production by all lymphocyte subsets showed a similar pattern at both time points. The CD56^hi^ and CD56^low^ NK cells were the dominant lymphocyte subsets producing the most of IFN-γ (Figure 8B and 8C). When PBMC from healthy controls were treated with HD IL-2 for 6 h, about 0.75% (range: 0.563-1.4%) CD4^+^ T cells and 1.82% (range: 1.3-3.65%) CD8^+^ T cells expressed IFN-γ, in contrast, 17.70% (range: 7.48-56.00%) CD56^hi^ NK cells and 8.86% (range: 5.28-24.00%) CD56^low^ NK cells secreted IFN-γ (Figure 8B). Importantly, at both time points tested, the CD56^hi^ NK cell subset (the main IFN-γ producing NK cell subset) showed defective secretion of IFN-γ in patients, whereas the IFN-γ release by CD4^+^ T and CD8^+^ T cells in patients and normal controls were comparable (Figure 8B and 8C). As for the CD56^low^ NK cells, we observed a significantly reduced IFN-γ-expressing cells after 6 h of HD IL-2 stimulation and a trend of reduction after 14 h of treatment. Taken together, our data suggest that an impaired IFN-γ secretion by NK cells was responsible for the defective STAT1 activation in NK and T lymphocytes from metastatic melanoma patient in response to IL-2.

## DISCUSSION

Dysfunctions in the immune system in cancer patients have been reported in many different cancer types [25–27, 29, 37]. These defects negatively affect the efficacy of cancer immunotherapies by reducing the responsiveness of immune cells. This could explain the relatively low response rates seen in metastatic melanoma patients in response to HD IL-2. In contrast to previous findings by Mortarini *et al*., our findings here found that STAT1 but not STAT5 activation was defective in PBMC from patients with metastatic melanoma in response to HD IL-2 [28]. In our study, we have used HD IL-2 with 6,000 IU/ml to better mimic the peak IL-2 concentrations measured in the serum of bolus HD IL-2 treated patients [38], while other studies used significantly lower of IL-2 concentrations to assess STAT activation [28, 39, 40]. We also titrated the IL-2 dose in initial experiments to make sure we were using a dose that saturated the normal STAT response in normal donors. Therefore, the findings we have observed here may eventually be more relevant and related to the clinical setting. Furthermore, for the first time we measured the STAT5 response in real-time in blood samples taken immediately before and 10 min into the first bolus IL-2 infusion in a series of patients receiving HD IL-2 for the first time. These experiments also indicated that STAT5 was dramatically activated to high levels in all patients tested, especially in NK cells where nearly all the cells exhibited pSTAT5 staining. Thus, these data support the notion that STAT5 activation is relatively normal in melanoma patients.

Another key finding in our study was that although pSTAT5 was directly activated by HD IL-2, STAT1 activation was indirectly through IFN-γ released by NK cells in response to HD IL-2. This was evidenced by a reduced proportion of pSTAT1 activation after IFN-γ neutralization in PBMC stimulated with HD IL-2. Our data also clearly suggest that NK cells were the dominant population secreting IFN-γ in response to IL-2 stimulation and a dysfunction in the ability of NK cells (especially the CD56^hi^ subset) from melanoma patients to secrete IFN-γ significantly contributed to the impaired pSTAT1 activation in all subsets. We also found that this impairment of STAT1 phosphorylation in lymphocyte subsets in patients occurred regardless of type of prior therapy each patient received before HD IL-2 treatment making it unlikely that it was due to an effect of prior treatment. Moreover, an intrinsic rather than therapy-induced impairment of IFN signaling has been recently described in lymphocytes from breast cancer patients [26]. This further suggests that an intrinsic IFN signaling defect could contribute to a poor responsiveness to IL-2 in melanoma patients.

Interestingly, we also found that the defect in STAT1 activation in CD4^+^ T cells and NK cell subsets in the patients with metastatic melanoma was age-related, while normal donors had a stable response with age. This decreased STAT activation was not associated with tumor burden or an overall shift in the median age or age range of the population relative to the normal donor pool. This finding suggests that signal transduction cascades of T cells and NK cells in aged-patients are altered substantially and could strongly affect the efficiency of overall immune response to cancer immunotherapies. It is not clear whether this aged-associated alteration occurs prior to or after disease development. Further study is needed to address this issue and could provide more insights into understanding why NK cells in particular develop this defective response with age in cancer patients and the molecular mechanisms behind this. NK cell defects have been noted in other types of cancer patients, such as head and neck squamous cell carcinoma patients [41–43]. In addition, differences in the loss of blood NK cell levels and NK cell function with age have been noted in the human population, with individuals have pre-mature loss of function being associated with immune dysfunction and increased morbidity. Interestingly, a study has also found an association between an improved maintenance of NK cell activity and the longer survival of centenarians (the oldest old) in the human population [44]. As cancer is a disease of aging and associated immune dysfunction, our finding here showing an accelerated loss of pSTAT1 activation in response to IL-2 in the melanoma patients may relate to this differential loss of NK activity in the normal human population making some individuals more prone to cancer and/or less able to control cancer progression through immune-mediated mechanisms. Due to limited blood samples from patients responded to HD IL-2, we were unable to compare how the STAT1 defects relate to the clinical response of HD IL-2 therapy in this study. However, further study is warranted to analyse whether the defects of STAT1 signaling are observed in HD IL-2 treated melanoma patients who progressed from disease in comparison to patients who responded to IL-2 therapy. Moreover, it would be interesting to further analyse how the defect of STAT1 signaling in immune cells may influence the clinical response in patients who undergo other treatment such as checkpoint blockade immunotherapies. Previous reports have shown several defects in type I and type II IFN signaling pathways in melanoma patients [29, 30]. It has been shown that STAT1 phosphorylation was significantly decreased in melanoma patients compared with healthy controls when stimulated with IFN-α [30]. A study by Critchley-Thorne *et al.* suggested that a downstream type-I IFN signaling was impaired in T and B cells from melanoma patients, while downstream type-II IFN signaling was only reduced in B cells of patients [29]. These findings could explain one mechanism of defective pSTAT1 activation when IFN-γ secretion in response to HD IL-2 was normal. However, in our study, we showed that inhibition of IFN-γ secretion itself could also contribute to abnormal STAT1 activation in melanoma patients, because STAT1 was not directly phosphorylated by IL-2 signaling, but depended on IFN-γ released by NK and T lymphocytes when stimulated with IL-2. These defects in IFN-γ response could be one of many other defects in the immune abnormalities, such as impaired IFN and STAT signaling in lymphocytes [28–30], diminished TCR-ζ chain [24, 32], reduced expression of p56^lck^ and Zap-70 in T cells [24], increased CD66a expression on NK cells [45] and their impeded cytotoxic functions [27].

It is known that IL-2 can regulate at least two different signaling pathways through the JAK-STAT, PI3K and MAPK pathways [7]. In our study, we showed that secretion of several cytokines by NK and T lymphocytes from patients in response to HD IL-2 was impaired, while Ki67 staining, which correlated to proliferation of those lymphocytes except CD8^+^ T cells, was similar as in normal controls. This is consistent with our data that STAT5 activation was not dramatically reduced since IL-2 induced STAT5 is involved in triggering of proliferative responses, while STAT1 activation via IFNs is more associated with the activation of lymphocyte (NK and T-cell) effector function and in fact have an anti-proliferative role in the immune response [46–48]. Thus, the decreased pSTAT1 response to IL-2 represents a growing abnormal effector response in melanoma patients separate from a proliferative response induced by IL-2. This focus on effector responses could help us to pinpoint any aberrant genetic or epigenetic mechanisms involved in the lack of immunotherapy responses in many patients. Other defects might also be involved in the decreased IFN-γ secretion in response to IL-2, such as a down-regulation of p56^lck^ and ZAP70 expression [24], which both are associated with IL-2Rβ subunit and can regulate the activation of nuclear factor of activated T cells (NFAT), a regulator of cytokine secretion, downstream of the IL-2R [49].

The response to HD IL-2 could probably depend on the patient intrinsic immune system and the immunogenicity of individual tumors. As in some cases, we have observed similar STAT signaling response from melanoma patients in comparison to healthy donors but yet progress from the disease after HD IL-2 therapy. This may possibly due to lack of tumor immunogenicity and mutated antigens as well as presence of immunesuppressive mechanism that affects immune response. However, a key question remaining is the mechanism behind the abnormal NK cell response to IL-2 in terms of IFN-γ secretion, especially by the CD56^hi^ NK cell subset found in our study here. One mechanism could be mediated through TGF-β, which is elevated in many cancer patients, including melanoma patients [50, 51]. However, the inhibitory effects of TGF-β, as described in previous studies are so far contradictory [52–55]. Some studies have claimed that TGF-β could independently suppress NK cells [56], while others showed that the inhibition effect of TGF-β required the presence of myeloid derived suppressor cells [57]. A recent study suggested that STAT5 activation in melanoma patients may be impaired as a result of elevated TGF-β in the serum. However, our recent observations suggest that this may not be the case. Firstly, in a recent biomarker study on HD IL-2 patients, we have not found any association between serum TGF-β levels and clinical response to IL-2 (Radvanyi et al., unpublished observations). Secondly, we did not observe significant inhibition of STAT1 activation or IFN-γ secretion in normal donor PBMC pre-treated with TGF-β before IL-2 stimulation. On the other hand, cytokines such as IL-10, which is known to exert immunosuppressive effect on various effector cells, has been described to suppress IFN-γ release by NK cells. In our study, we did not find an increase in IL-10 secretion in PBMC from patients after HD IL-2 treatment. Indeed, there was decrease IL-10 level when PBMC were treated with HD-IL-2 and this further excluded the possibility of its contribution to the impairment of IFN-γ production by NK cells. These findings however do not exclude the possibility of other inhibitory molecules secreted by tumor cells and immune suppressor cells that may responsible for the defect we have found in the current study. For example, factors such as adiponectin [58] and immune-suppressive exosomes [59, 60] have been shown to have inhibitory effect on NK cells and other lymphocytes. Immunesuppressor cells such as Tregs and myeloid derived-suppressor cells (MDSC) may inhibit the IFN-γ production by NK and T cell population [61–63]. Tregs have been shown to inhibit IFN-γ by multiple pathways such as direct cell-to-cell contact as well as IL-10 and TGF-β productions [61, 62]. Tregs express constitutively higher levels of high affinity IL2Rαβγ and consume huge amount of IL-2, thus, deplete the availability of IL-2 to NK and T effector cells and affect their activation as well as IFN responsiveness. Moreover, increased expansion of Tregs have been shown in patients after HD IL-2 therapy [15] and this may contribute to the inhibition of IFN response. Thus, it is of interest to futher study whether Treg depletion by the application of IL-2 fusion protein or IL-2 analog that preferentially bind to the IL-2Rβγ to circumvent the inhibitory effect of Tregs may revert the impairment of IFN response. Of note, our recent study has found that IL-2 analog such as F42K can circumvent the expansion of highly suppressive ICOS^+^ Tregs and induce NK cell expansion as well as activation can have important impact for improving IL-2 therapy. In addition, Study by Mundy-Bosse et al has recently shown elevated levels of MDSC and the production of iNOS protein and nitric oxide by MDSC played a role in suppressing IFN response in mouse tumor model [63]. The same study also showed that depletion of MDSC with gemcitabine or an anti-GR1 antibody led to the restoration of IFN response. These studies suggest depletion of Tregs especially ICOS+ Tregs and MDSC may revert the poor IFN responsiveness in cancer patients and improved their response to HD IL-2 therapy. Furthermore, the relevance of these mechanisms to the dysregulation of immune activation observed in our current study will need to be explored in future studies.

## MATERIALS AND METHODS

### Reagents and antibodies

MSD^®^ 96-well Multi-Spot^®^ tissue culture kit was purchased from Meso Scale Discovery (MSD). Flourochrome-conjugated monoclonal antibodies (mAbs) against CD3, CD4, CD8, CD20, STAT1, pSTAT1-Y701, pSTAT5-Y694, Ki67, and IFN-γ were purchased from BD Biosciences (San Jose, CA). Anti-CD56 mAb (clone: N901) was purchased from Beckman Coulter (Pittsburg, PA). Anti-Foxp3 mAb and IFN-γ neutralizing mAb (clone: NIB42) were purchased from eBioscience (La Jolla, CA).

### Melanoma patient and healthy donor samples

Peripheral blood samples were obtained after informed consent within 2 weeks of starting IL-2 treatment from a total of 23 patients (14 males, 9 females with ages ranging from 29-69 years old; see Table S1 on-line), diagnosed with stage IV melanoma according to American Joint Committee Cancer (AJCC) criteria and admitted to University of Texas, MD Anderson Cancer Center, Houston, Texas. All blood samples from patients were collected at least two months after prior treatments, including surgery, radiotherapy, chemotherapy and biological therapy. In some cases, a blood sample was drawn immediately before the first bolus of IL-2 infusion and after 10 minutes of starting bolus HD IL-2 infusion (15 min total infusion time) for real-time analysis of STAT activation in whole blood. A summary of age, gender, disease and therapy of all patients are shown in Table S1 on-line. Intravenous blood samples from age and gender-matched normal donors (n=23) were obtained from Gulf Coast Regional Blood Center (Houston, Texas). All peripheral blood mononuclear cells (PBMC) used in this study were isolated by Ficoll density gradient centrifugation (HISTOPAQUE^®^-1077, Sigma-Aldrich, St. Louis, MO) and cryopreserved in 90% FBS (Atlanta biologicals) with 10% DMSO (Sigma-Aldrich, St. Louis, MO). The experiments were approved by the Institutional Review Board of University of Texas, MD Anderson Cancer Center.

### Cell culture

Cryopreserved PBMC from healthy controls and patients were thawed, washed and rested for 1 hour in Iscove's Modified Dulbecco's Medium Invitrogen-Gibco, Carlsbad, CA) supplemented with 1% human AB serum (Gemini, West Sacramento, CA). Then, 2 × 10^6^ PBMC were stimulated with or without 6,000 IU/ml IL-2 (Proleukin, Novartis, Morristown, NJ) for the indicated time points based on initial dose-response studies performed with normal donors (see Figure S1 on-line). Prior to flow cytometry staining, cell culture supernatants were collected after 24 h of IL-2 stimulation for MSD multiplex cytokine assay. For IFN-γ blocking assay, neutralizing anti-IFN-γ monoclonal antibody (mAb) was added together with IL-2 at a final concentration of 100 μg/ml. Cells were then harvested for pSTAT1 staining.

### Flow cytometry analysis

PBMC obtained from both melanoma patients and healthy donors stimulated with or without IL-2 were harvested, washed and surface stained for T-regulatory cells (Tregs) as described, CD4^+^ T cells, CD8^+^ T cells, CD56^hi^ and CD56^lo^ NK cell subsets using anti-CD3, anti-CD4, anti-CD8, anti-CD56 and anti-Foxp3 mAbs. Treg cell staining was performed using Foxp3 staining kit (eBioscience, La Jolla, CA) according to the manufacturer's instructions. To exclude B cells, PBMC were stained with anti-CD20 mAb and CD20^+^ B cells were excluded from the analysis. For STAT1, pSTAT1 and pSTAT5 phosphoflow staining, the HD IL-2 stimulated or non-stimulated PBMC or fresh whole blood samples obtained from melanoma patients before and 10 min after bolus of HD IL-2 infusion were first fixed with Cytofix buffer for 10 min (BD Biosciences, San Jose, CA) and permeabilized with phosflow Perm Buffer III (BD Biosciences) for another 20 min. Cells were washed and then stained for T- and NK-cell surface markers and intracellular STAT1, pSTAT1-Y701 or pSTAT5-Y694 for 30 min at room temperature. To estimate proliferation capacity, HD IL-2 stimulated and non-stimulated PBMC were stained for Ki67. For Ki67 intracellular staining, 2 × 10^6^ of PBMCs from patients and normal controls were stimulated with 6,000 IU/ml IL-2 for 4 days. Cells were harvested, washed and stained for surface markers to distinguish different lymphocyte subsets as above and then fixed and permeabilized using fix/perm buffer (eBiosciences La Jolla, CA) followed by Ki67 staining. Flow cytometry data were acquired using a FACSCanto II (Becton Dickinson, San Jose, CA) and analyzed with Flowjo version 7.6.1 (Treestar, Ashland, OR).

### Intracellular cytokine staining

To examine the capability of T- and NK-cell subsets to produce IFN-γ, 2 × 10^6^ of PBMCs were seeded in 96-well plate and stimulated with 6,000 IU/ml IL-2 for 6 h or 14 h. GolgiStop (BD Biosciences, San Jose, CA) was added at the concentration recommended by manufacturer during the last 5 h of incubation. Cells were then harvested, washed and stained for T- and NK-cell surface markers. The stained cells were then fixed and permeabilised with Cytofix/Cytoperm according to manufacturer's instruction (BD Biosciences, San Jose, CA) and stained with IFN-γ mAb.

### MSD multiplex cytokine assay

The levels of different cytokines including GM-CSF, IL-1β, IL-6, IL-8, IL-10, IL-12p70, IFN-γ and TNF-α in the culture supernatants were measured using an MSD^®^ 96-well Multi-Spot^®^ tissue culture kit according to the manufacturer's instructions. Briefly, samples and calibrators were incubated in pre-coated MSD plates with vigorous shaking at room temperature for 2 hours. Then, detection antibody was incubated after washing for another 2 h. After addition of read buffer T, the data were acquired and analyzed using the SECTOR imager 2400 (Meso Scale Discovery, Gaithersburg, MD).

### Statistical analysis

Data were analyzed for significant differences using a two-sided Mann-Whitney test, Spearman correlation and ANOVA test where appropriate and as indicated. A value of *p* < 0.05 was considered significant. All analyses were performed with Graphpad Prism 5 software (GraphPad, San Diego, CA).

## SUPPLEMENTARY FIGURES AND TABLES


