# Supplementary material for: Defective STAT1 activation associated with impaired IFN-γ production in NK and T lymphocytes from metastatic melanoma patients treated with IL-2
Source: Oncotarget. 2016 Apr 11;7(24):36074–91. doi: 10.18632/oncotarget.8683 (PMC5094984; doi:10.18632/oncotarget.8683)
Supplement: Supplementary file 1 [file oncotarget-07-36074-s001.pdf]

Defective STAT1 activation associated with impaired IFN-γ production in NK and T lymphocytes from metastatic melanoma patients treated with IL-2

SUPPLEMENTARY FIGURES AND TABLES

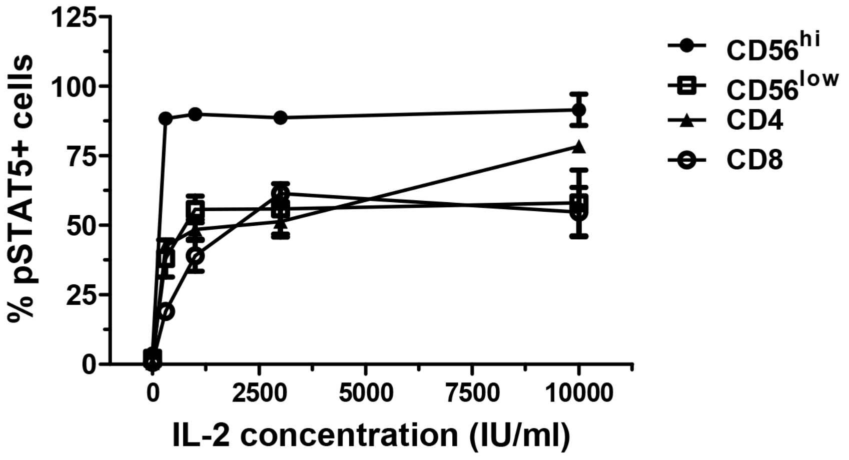

Supplementary Figure S1: Dose-dependent STAT5 phosphorylation induced by IL-2 and CD4^{+}T, CD8^{+} T, CD56^{hi} and CD56^{lo} NK subsets. A. Different doses of Proleukin® IL-2 at 0, 300, 1000, 3000 and 10000 IU/ml were added to stimulate PBMC (2 x 10^{6}) from three healthy controls for determining the optimal dose of IL-2 that induced pSTAT5 expression in different CD4^{+}T, CD8^{+}, CD56^{hi} and CD56^{lo} NK subsets. Phospho-STAT5 was detected by phospho flow staining and IL-2 at 6000 IU/ml was found to be the optimal dose for inducing maximal STAT5 activation in all of the lymphocyte subsets being analysed after 24 h of stimulation.

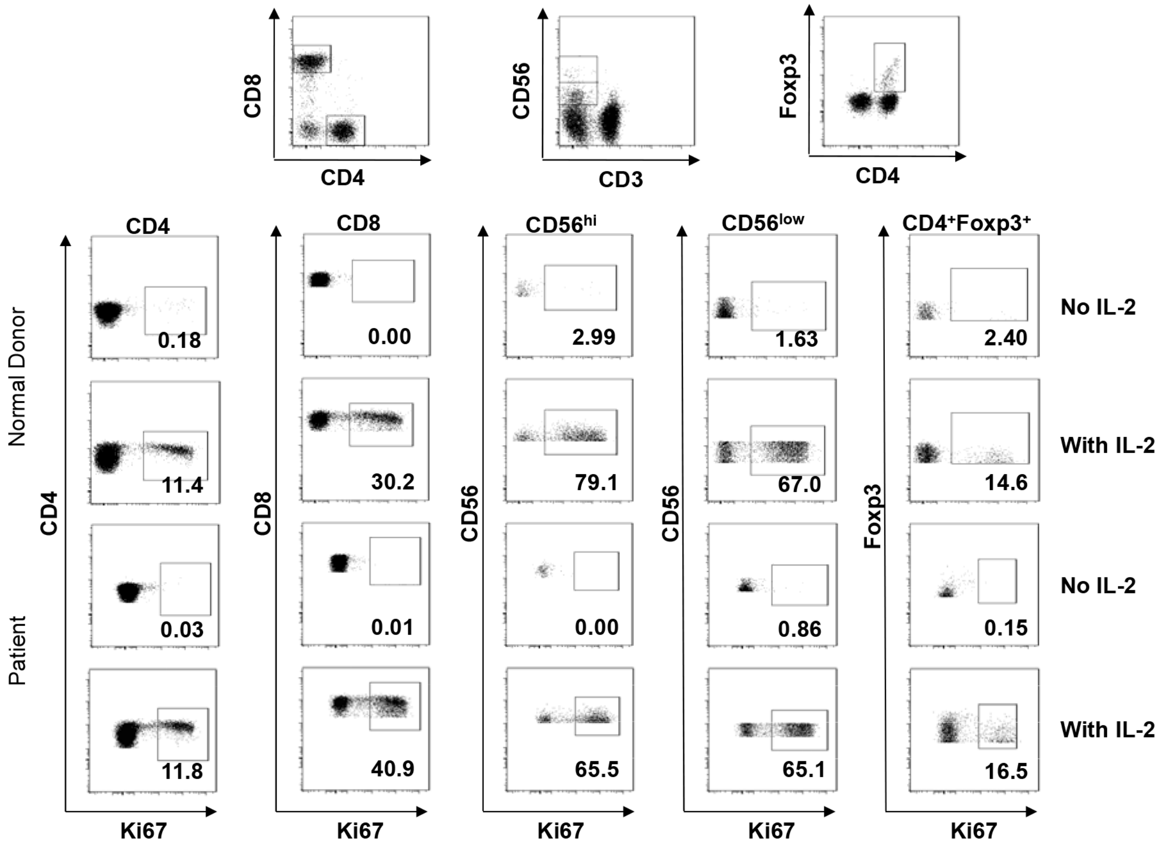

Supplementary Figure S2: Proliferation capacity of T and NK cell subsets in response to HD IL-2 stimulation. Intracellular Ki67 staining was performed on PBMC isolated from healthy controls and patients after 24 h of HD IL-2 (6,000 IU/ml) stimulation. Flow cytometry data indicate a similar proliferation capacity in all T and NK cell subsets as measured by Ki67 expression.

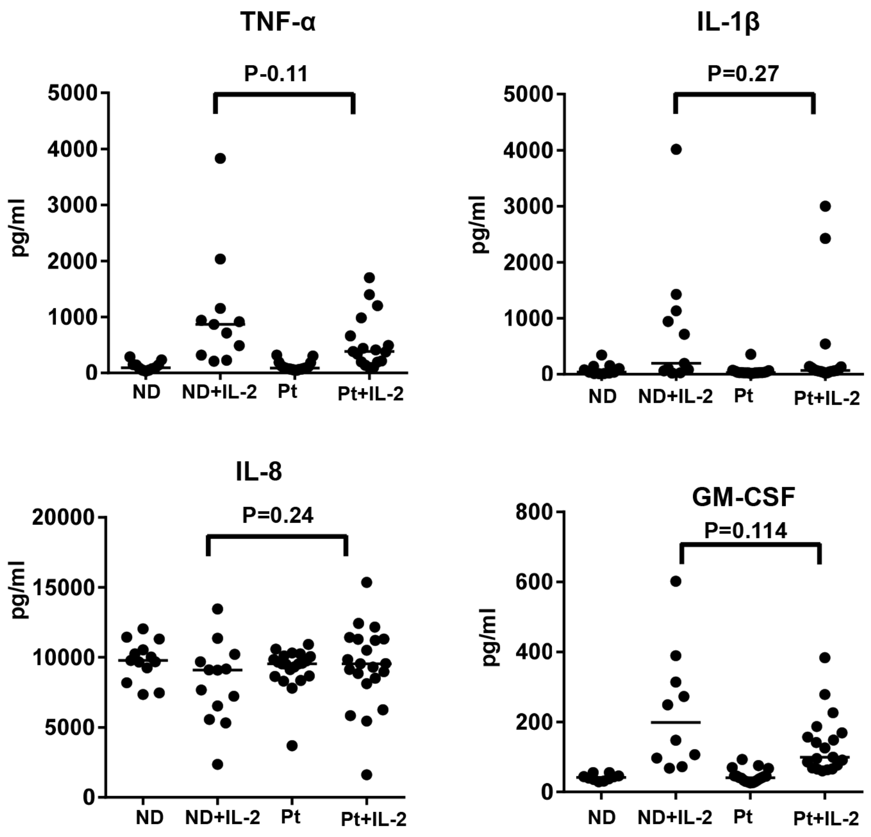

Supplementary Figure S3: Measurement of cytokine secretions by HD IL-2 stimulated PBMC. Results of cytokine secretion show a similar release of TNF-α, IL-1β, IL-8 and GM-CSF between melanoma patients (Pt n=20) and healthy donors (ND, n=15) when PBMC were stimulated with 6000 IU/ml IL-2 for 24 h. Medians in the scatter plots are indicated by the horizontal bars.

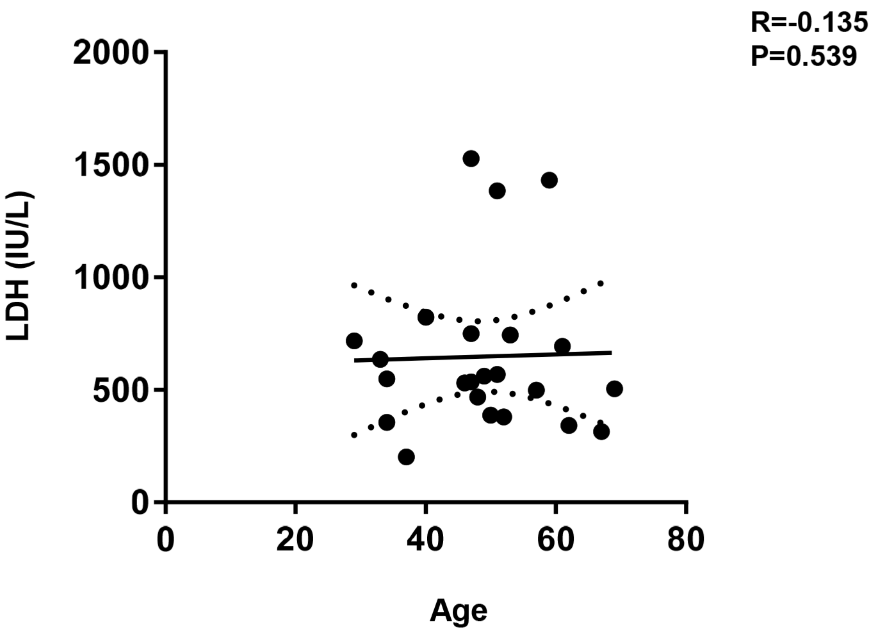

Supplementary Figure S4: Tumor burden is not correlated with age. Serum levels of lactate dehydrogenase (LDH) were measured prior to HD IL-2 therapy in patients with melanoma. Spearman correlation test was used to compare LDH values from cancer patients with age. $P < 0.05$ was considered as significant.

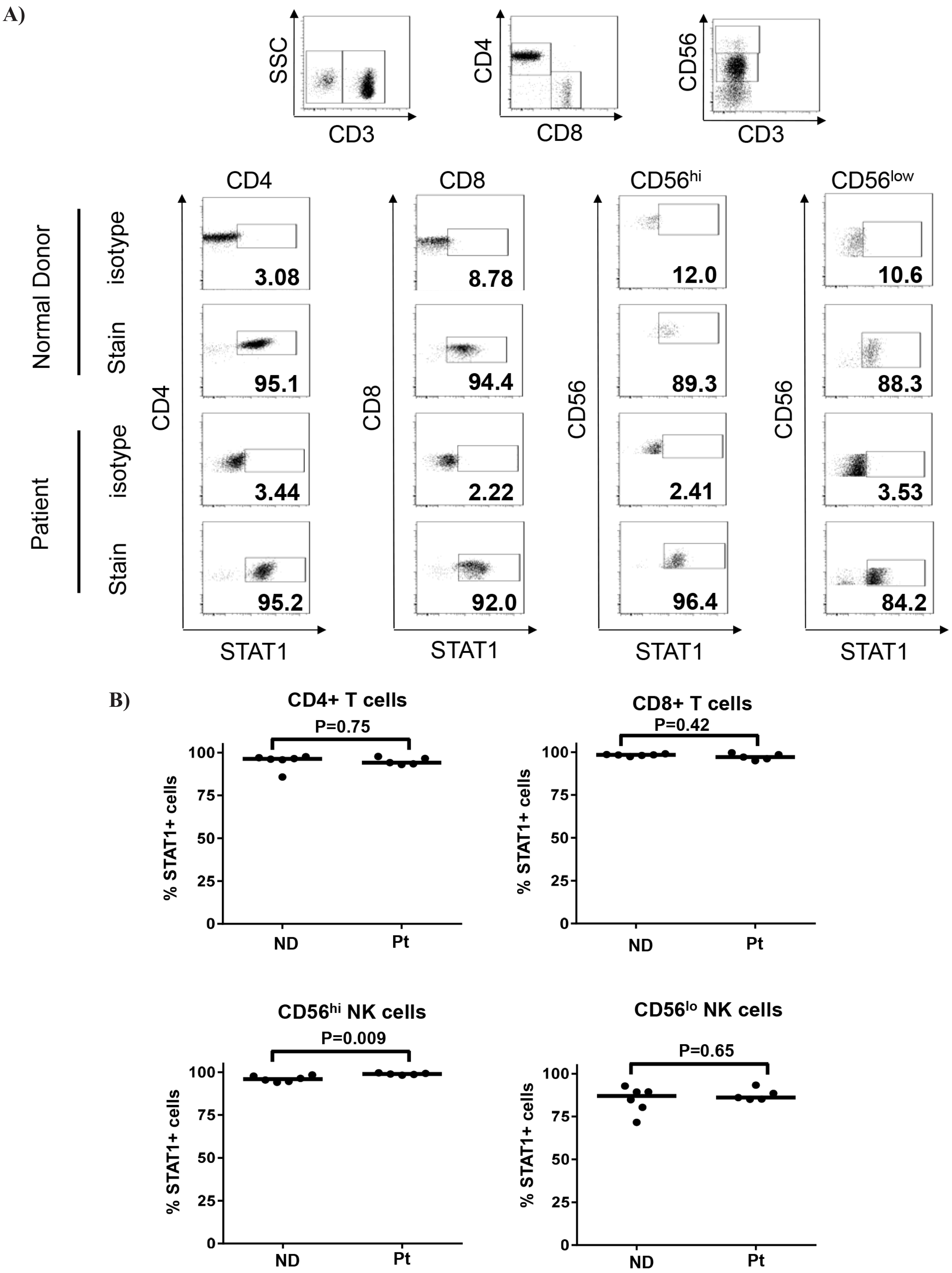

Supplementary Figure S5: Total STAT1 expression by T and NK cell subsets. PBMC stimulated with or without HD-IL2 (6000 IU/ml) for 24 h were stained for STAT1. **A.** Gating strategy for identifying different T and NK cell subsets are shown. **B.** The analysis of proportion of cells expressing total STAT1 in the indicated cell subsets in normal controls (ND) and patients (Pt). $P < 0.05$ was considered as significant.

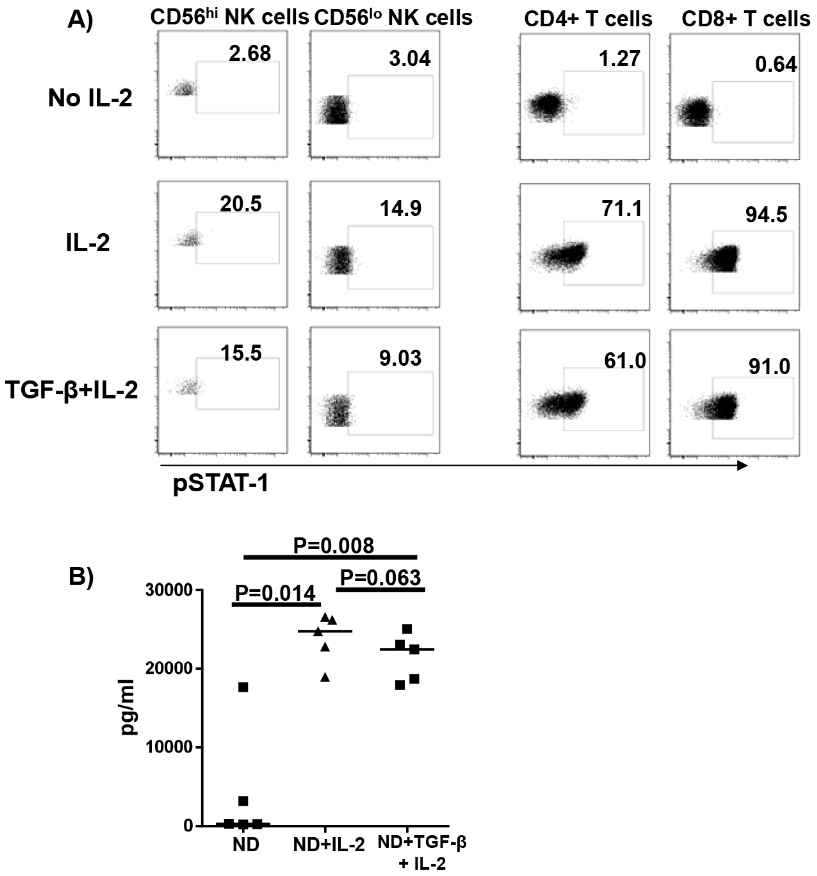

Supplementary Figure S6: Effect of TGF-β on STAT1 activation and IFN-γ secretion in IL-2 treated T and NK cells. PBMCs from healthy controls (N=5) were pre-treated with TGF-β (1 ng/ml) and incubated for 48 h prior to the addition of IL-2 (6000 IU/ml) for another 24 h incubation. **A.** The flow cytometry dot plots show the effect of TGF-β on STAT1 activation in different T and NK cell subsets. **B.** IFN-γ secretion in the presence or absence of TGF-β was measured.

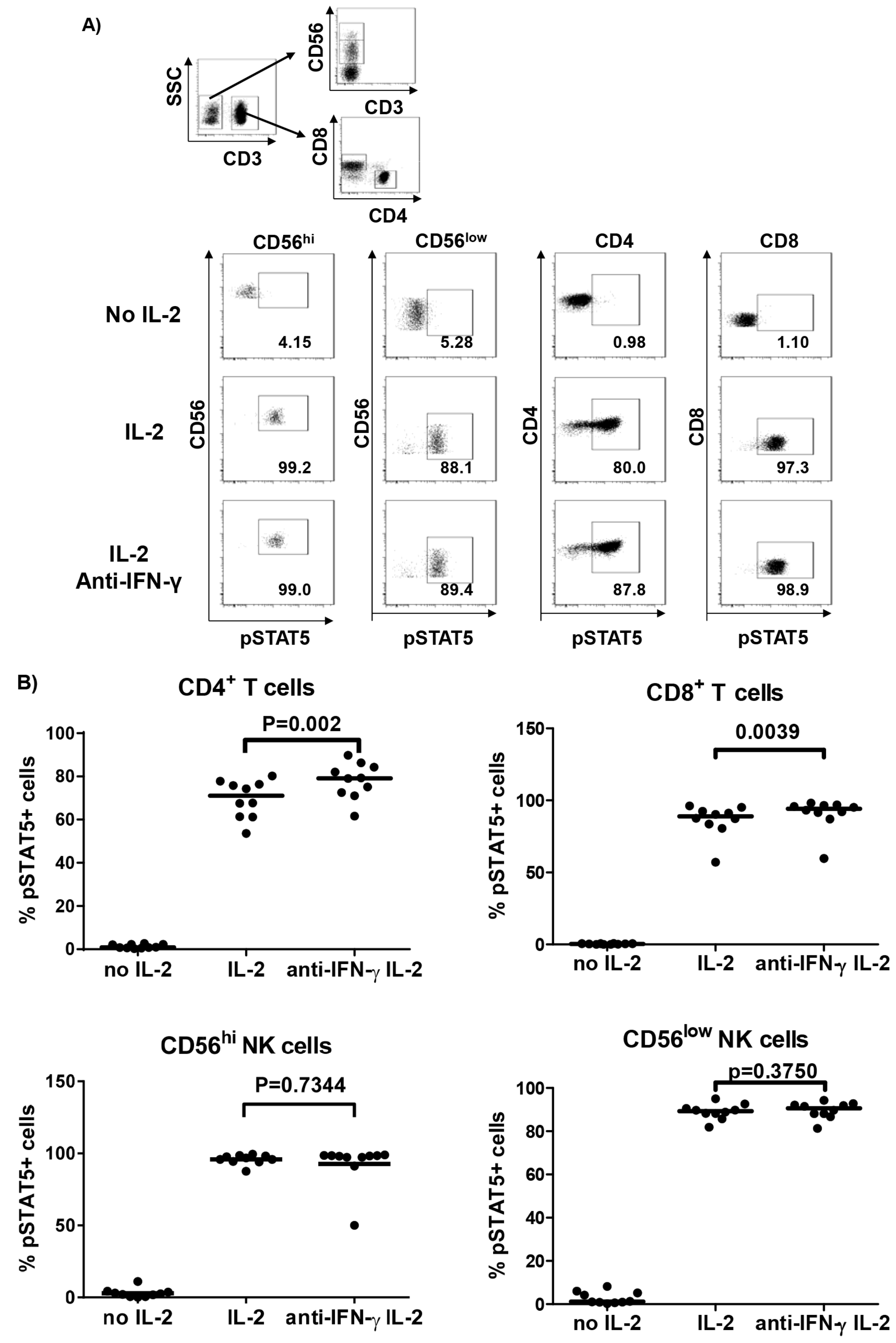

Supplementary Figure S7: Effect of IFN-γ blockade on STAT5 activation in T-cell and NK cell subsets. A. The flow cytometry dot plots show blockade of IFN-γ secretion using neutralizing anti-IFN-γ mAb (100 μg/ml) that was added together with IL-2 (6000 IU/ml) for 24 h. The blockade of IFN-γ secretion did not significantly cause a reduction in the frequency of pSTAT5^{+} cells in the T and NK cell subsets. **B.** The percentage of IL-2 induced pSTAT5 in CD4^{+}, CD8^{+} T cells, CD56^{hi} and CD56^{lo} NK cells as measured by flow cytometry.

Table S1: Clinical Information of Patient

Sample ID	Age	Sex	Therapy within last 2 months	Metastases	Stage
P6	59	M	Chemotherapy, radiation therapy, surgery	Bilateral lung, LN, subcutaneous-back, breast, shoulder	IV
P7	69	M	Surgery	Mediastinum, liver, spleen, intramuscular lesions	IV
P9	67	M	Adjuvant IFN therapy, Surgery	Inguinal LN, hip lesions	IIIc
P16	53	M	Surgery	Lung, mediastinum, inguinal LN, subcutaneous, knee	IV
P17	48	F	Surgery, adjuvant IFN therapy	Shoulder, back, cheek	IV
P20	61	M	Surgery	Scalp, scapula, liver, neck, periauricular	IV
P25	37	F	Surgery	Lung	IV
P26	51	M	Surgery, radiation therapy	Spine, lung, pancreas, adrenal, perinephric	IV
P30	57	M	IFN adjuvant therapy, GP100 vaccine adjuvant therapy, CR011-vcMMAE, surgery	Neck, mediastinal LN	IV
P31	49	F	Surgery, DTIC, radiation	Lungs, liver, inferior vena cava	IV
P36	51	F	Surgery, adjuvant IFN therapy, isolated limb perfusion with Melphalan	Cutaneous and subcutaneous in-transit melanomas on lower extremities	IIIc
P37	46	F	Adjuvant IFN therapy,	Lung	IV
P38	47	F	Surgery, radiation therapy	Subcutaneous tissue, LN (bilateral breast, axilla, jips, scapula)	IV
P40	47	M	Surgery	Neck, spleen, LN	IV
P46	47	F	Surgery, adjuvant IFN therapy	Arm, breast, lung, spleen, retroperitoneum, duodenum, subcutaneous tissue	IV
P47	29	M	Surgery, adjuvant IFN therapy	Neck, LN, lung	IV
P48	34	F	Surgery, radiation therapy	Scalp (skin)	IV
P49	50	F	Surgery, adjuvant IFN therapy	Liver, pelvic LN	IV
P50	62	M	Surgery, radiation therapy, adjuvant IFN therapy	Bilateral lung, scapula	IV
P52	33	M	Surgery, melphalan and ADH, radiation therapy, Sorafenib and Temsirolimus	Leg	IV
P53	40	M	Surgery, adjuvant IFN therapy, Nexavar and Torisel, carboplatin and Taxol	Scapula, femur	IV
P54	34	M	IFN therapy	Lung, axilla	IV
P56	52	M	Radiation	LN, thigh, chest wall, mesenteric, & submandibular lesions	IV

Table S2: Gender and age of melanoma patient and normal donor test samples

Variable		Type of sample		Total (N)	p-value[a]
		Patients	Normal donors		
Gender	Male	14	12	26	0.767
	Female	9	11	20	

[a]Statistical analysis: Fisher exact test

Table S3: Associations between age and type of samples

Variable	Patients			Normal donors			p-value[b]
	Median	Min	Max	Median	Min	Max	
Age (years)	49	29	69	43	24	66	0.1273

[b]Statistical analysis: Mann-Whitney test
